# Microenvironmental Factors Modulating Tumor Lipid Metabolism: Paving the Way to Better Antitumoral Therapy

**DOI:** 10.3389/fonc.2021.777273

**Published:** 2021-11-23

**Authors:** Limeng Cai, Minfeng Ying, Hao Wu

**Affiliations:** Cancer Institute (Key Laboratory for Cancer Intervention and Prevention, China National Ministry of Education, Zhejiang Provincial Key Laboratory of Molecular Biology in Medical Sciences), The Second Affiliated Hospital, Zhejiang University School of Medicine, Hangzhou, China

**Keywords:** tumor micoenvironment, metabolic reprogramming, microenvironment factor, cancer therapy, lipid metabolism

## Abstract

Metabolic reprogramming is one of the emerging hallmarks of cancer and is driven by both the oncogenic mutations and challenging microenvironment. To satisfy the demands of energy and biomass for rapid proliferation, the metabolism of various nutrients in tumor cells undergoes important changes, among which the aberrant lipid metabolism has gained increasing attention in facilitating tumor development and metastasis in the past few years. Obstacles emerged in the aspect of application of targeting lipid metabolism for tumor therapy, due to lacking of comprehensive understanding on its regulating mechanism. Tumor cells closely interact with stromal niche, which highly contributes to metabolic rewiring of critical nutrients in cancer cells. This fact makes the impact of microenvironment on tumor lipid metabolism a topic of renewed interest. Abundant evidence has shown that many factors existing in the tumor microenvironment can rewire multiple signaling pathways and proteins involved in lipid metabolic pathways of cancer cells. Hence in this review, we summarized the recent progress on the understanding of microenvironmental factors regulating tumor lipid metabolism, and discuss the potential of modulating lipid metabolism as an anticancer approach.

## 1 Introduction

The increased metabolic needs of tumor cells in a setting of hypoxia, acidosis and nutrient deprivation, highlight the importance of metabolic reprogramming to support rapid proliferation, continued growth, invasion, metastasis and resistance to therapeutic interventions (1). The metabolism of critical nutrients like glucose and glutamine undergoes crucial remodeling in tumors, which thousands of studies have shed light on. Altered lipid metabolism is another remarkable feature of tumor metabolism and has received renewed interest recently guiding response and resistance to antitumoral therapies (2). Lipids encompass a diverse group of hydrophobic biomolecules including phospholipids, triacylglycerols, and sterols, mostly composed of common building blocks like fatty acids (FAs) and cholesterol. They actively participate in a variety of biological processes (2). Aside from being essential structural components of membranes, lipids also function as energy sources and metabolic substrates, thereby highly needed by tumor cells to facilitate rapid proliferation (2). Particularly, lipids appear to be more important in non-glycolytic tumors such as prostate cancer that mainly rely on fatty acid oxidation (FAO) rather than glycolysis for ATP production (3–5). The role of lipids in the synthesis of pro-tumorigenic signaling molecules is also worth to be addressed. Signaling lipids, such as phosphatidylinositol and lysophosphatidic acid (LPA), exert oncogenic effects by activating signaling pathways driving proliferation and migration (6, 7).

Invasion and metastasis are the deadliest aspects of tumor, while lipid metabolism also has an established role in fueling these processes. For instance, cholesterol is the critical component of lipid rafts, which are small lipid domains within the cell membrane and mediate signal transduction and plasma membrane protein sorting. Lowering cholesterol level disrupts lipid rafts and then inhibit cell migration by inducing CD44 shedding, which is the principal cell adhesion receptor expressed in tumor cells (8). EGFR signaling, the vital signaling pathway in tumor migration, is also disturbed when destroying the structure of lipid rafts (9). Besides, various dysregulated enzymes in lipid synthesis and catalysis are well-elucidated to promote metastasis of tumor cells (10). Also, CD36-mediated lipid metabolism is found to be a significant feature in metastasis-initiating cells in oral squamous cell carcinoma (11). Therefore, lipid metabolism is increasingly recognized as a critical culprit in the pathogenesis of malignancies, and its regulation represents a fertile field of research. To target this metabolism in tumor cells specifically, it is important to identify regulators and relevant mechanism governing lipid metabolic reprogramming in malignant cells.

While oncogenic mutations have displayed pleiotropic effects in rewiring lipid metabolism to promote carcinogenesis, little is known about the contribution of tumor microenvironment (TME) in this process (12, 13). Solid tumor cells are surrounded by the neighboring cellular (e.g., stromal cells and infiltrated immune cells) and molecular components (e.g., cytokines, metabolites, exosomes and extracellular matrix), which are described as TME. Tumor cells closely interact with adjacent niche and take advantage of it, thereby sustain escaping strategies to thrive in the challenging environment (14). Similarly, TME in turn highly contributes to metabolic rewiring of critical substrates in cancer cells and its impact on lipid metabolism has become a topic of renewed interest (15). As the lipid metabolism of cancer cells has been extensively studied and well-documented (2), in the following sections, we will mainly focus on the recent revealed interaction of TME factors on the lipid metabolism of cancer cells while first briefly revisit the alteration of tumoral lipid metabolism for a better understanding.

## 2 Alterations of Lipid Metabolism in Tumor Cells

Increased scavenging for extracellular lipids and *de novo* synthesis are important routes for tumors to acquire lipids. Cell surface receptors and transporters for lipids are thereby upregulated in various tumors, such as CD36 and low-density lipoprotein receptor (LDLr), as well as FA binding protein (FABP) and FA transport protein (FATP) (16–18). For instance, CD36 is significantly upregulated in malignant epidermal tumor cells such as ovarian cancer (19) and gastric cancer (20), and is correlated with metastasis and poor prognosis for patients. Higher LDLr expression has been reported in pancreatic adenocarcinoma (PDAC), glioblastoma, triple negative and HER2 overexpressing breast cancers for higher uptake of cholesterol (21–23). But it’s downregulated in hepatocellular carcinoma while *de novo* cholesterol biosynthesis is elevated (24).

Various tumors exhibit a phenotype of exacerbated *de novo* lipogenesis, irrespective of the levels of extracellular lipids (25). Acetyl-CoA is the main substrate for lipid synthesis and enhanced glucose and glutamine metabolism could contribute to this metabolic pathway by providing lipogenesis precursors like citrate through tricarboxylic acid cycle (TCA) (25). Moreover, various enzymes involved in *de novo* lipogenesis pathway are significantly up-regulated in cancers, such as acetyl-CoA carboxylase (ACC) and fatty acid synthase (FASN) in FA synthesis and 3-hydroxy-3-methylglutaryl-CoA reductase (HMGCR) in cholesterol synthesis (26). These key enzymes represent appealing therapeutic targets for blocking lipid metabolism and has been extensively investigated for cancer therapy (26, 27).

The primary products of *de novo* FA synthesis are saturated FA (SFA), which could be desaturated to monounsaturated FA (MUFA) by stearoyl-CoA desaturase (SCD). MUFA is the key substrates in the formation of phospholipids, cholesteryl esters and triglycerides in cell membranes including the endoplasmic reticulum (ER) membrane, thus the imbalance of SFA/MUFA or accumulation of SFA will lead to can reduce membrane fluidity and dynamics, causing disrupted signaling and impaired ER homeostasis (28, 29). SCD1 is found to be overexpressed in many types of cancers, such as breast, ovarian, lung, gastric, colon cancers, hepatocellular carcinoma (HCC) and clear-cell renal cell carcinoma (ccRCC) (30–36), and endows cancers with progressive, invasive and migratory properties, which makes SCD1 a promising anti-cancer target. Being responsible for the biosynthesis of the phosphatidylcholine (the major phospholipid in cell membranes), the Kennedy pathway also shows great significance in tumors and provides appealing molecular targets for anti-cancer therapies, particularly the choline kinase (Chk), the initial enzyme in the Kennedy pathway (37). Overexpression of Chk is observed in human breast, ovarian, colorectal, lung, prostate and liver cancers (38–41). High levels of choline phospholipid metabolites and Chk are found to play a crucial role in tumorigenesis, progression and therapy resistance (37–39, 42).

Excessive lipids are then usually stored in lipid droplets (LDs) as neutral lipids (mainly triglycerides (TGs) and cholesterol esters (CEs)). Indeed, LD accumulation is widely observed in many human malignancies such as prostate cancer (43), glioblastoma (44) and ovarian cancer (45). It’s increasingly considered as an emerging hallmark of cancer aggressiveness, especially in tumor cells exposed to nutrient deprivation and hypoxia (46, 47). Notably, cancers are reported to employ LDs as means to promote cancer cell growth and proliferation such as central anti-lipotoxic organelle, regulation of FA trafficking and distribution, maintenance of ER and regulation of autophagy (46). Based on the evidence that LD accumulation causes chemotherapy resistance *via* inhibiting drug-induced apoptosis (48), spectroscopy imaging tools such as fourier transform infrared spectroscopy and Raman spectroscopy have promising roles in predicting therapy outcome in patients (48–50).

Interestingly, though FA oxidation and FA synthesis (FAS) are used to be considered incompatible on account of the suppressive effect of malonyl-CoA (a fatty acid synthesis intermediate) on FAO, there have been studies countering this argument in leukemia and breast cancers (51, 52) and findings in acidic-adapted cancer cells proved that the concomitance of FAO and FAS were allowed by histone deacetylation-mediated acetyl-CoA carboxylase 2 (ACC2) inhibition (51–53). Lipid degradation not only provides metabolic fuels for cancer cells through mitochondrial fatty acid β-oxidation (FAO), but also generates acetyl-CoA for lipogenesis and provides the critical reductive force NADPH. Yet FAO rewiring and its role in tumor have only recently been heeded. Acute myeloid leukemia, glioma, triple-negative breast cancer, K-Ras mutant lung cancer, and hepatitis B-induced HCC exhibit high rates of FAO (54–58) while some non-glycolytic cancers such as prostate tumor and diffuse large B-cell lymphoma, preferentially use FAO as the dominant bioenergetic pathway for survival (3, 59). Overexpressed FAO enzymes are also found in cancer cells show high activity of FAO, such as rate-limiting enzyme carnitinepalmitoyl transferase 1 (CPT1) (60–62).

The regulation of lipid metabolism involves a complex transduction signaling pathways. Generally, the enhanced lipogenesis in many cancer cells (such as glioblastoma, breast and ovarian cancer) are likely consequences of increased expression of the pivotal transcription factors, sterol regulatory-element binding proteins (SREBPs) (63). It’s well established that SREBPs play distinctive roles in modulating the genes involved in the uptake and synthesis of fatty acid and cholesterol while inhibiting SREBPs is detrimental to cancer cell (64–66). As important nodes of convergence and divergence within biological signaling networks, SREBPs respond to the upstream signaling [i.e., PI3K/AKT/mTOR, AMPK pathway (67, 68)] and to changing nutritional status in the TME. Other pathways like peroxisome proliferator-activated receptors (PPARs) and liver X receptors (LXRs) signal transduction pathway also play an active part in modulating lipid reprogramming, which are both ligand-activated nuclear transcription factors. Investigation of their roles in carcinogenesis has gained momentum (69). PPARα and PPARβ/δ induce lipid oxidation, while PPARγ activates lipid storage and adipogenesis. LXR pathway is critical in maintaining cholesterol homeostasis and is usually impaired in cancer with elevated cholesterol level, suggesting an antitumor effect exerted by LXR activation (70). These pathways form a fine regulatory network and tangle with each other, emphasizing the necessity of carefully consideration in targeting at upstream or downstream genes.

**Figure 1:**
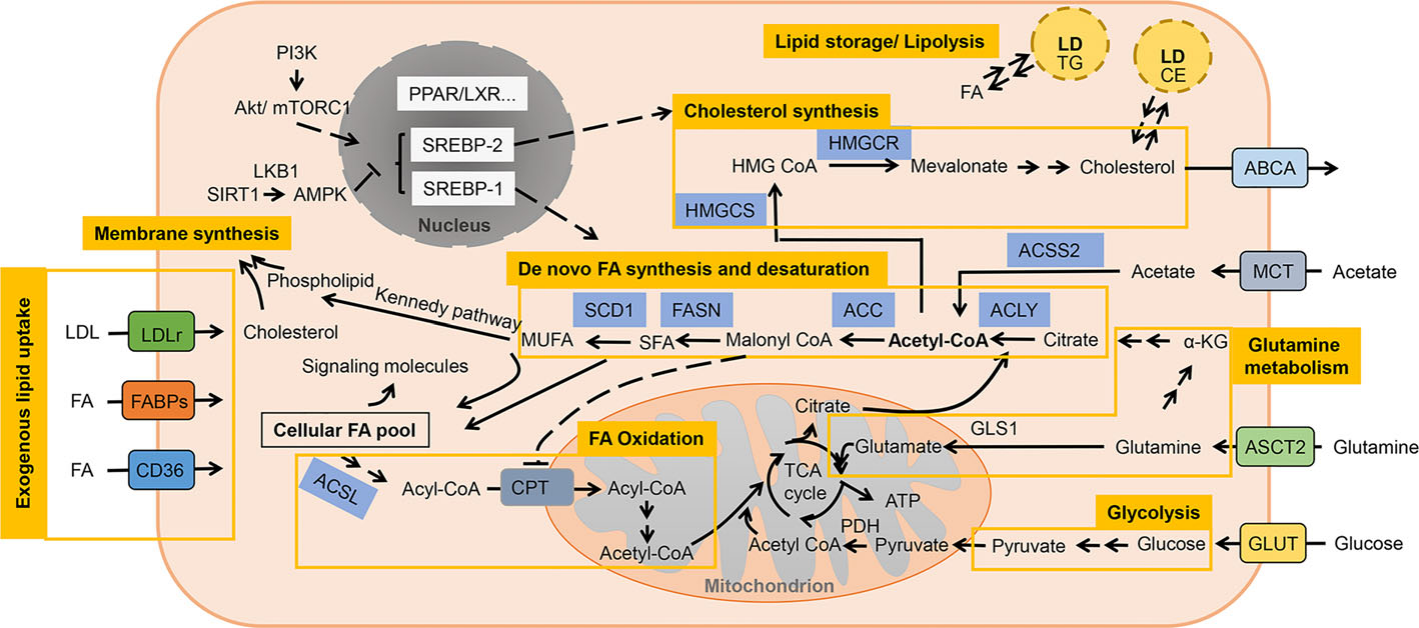
A brief representation of altered lipid metabolism in cancer cells. Lipids are metabolized in cancer cells involving molecular process of uptake, *de novo* synthesis, degradation, storage and lipolysis. Aside from being structural components of membrane, lipids also function as energy sources and signaling factors coordinating various biological processes. Acetyl-CoA is the main substrate for lipid synthesis while the altered metabolism of glucose, glutamine and acetate could contribute to this metabolic pathway by providing precursors. FAs are then either break down through mitochondrial β-oxidation to produce energy, or utilized in membrane synthesis together with cholesterol. Excessive lipids are then usually stored in LDs as TG and CE, and could be mobilized for another source of lipids. LD is recognized as important cellular organelle in regulating autophagy and anti-lipotoxicity. SREBPs are pivotal transcription factors that respectively modulates the genes involved in the uptake and synthesis of fatty acid and cholesterol, and responds to the upstream signaling (i.e., PI3K/AKT/mTOR, AMPK pathway). Other pathway like PPAR and LXR signaling also play an active role in modulating lipid reprogramming.

Given the metabolic alterations made in lipids being a pivotal element defining tumor cell fate (**Figure 1**), understanding how it is remodeled and verifying regulating factors might provide us a tantalizing therapeutic target.

## 3 Microenvironmental Factors

With the focus interest of anti-tumor therapy shifted from a tumor cell-centered viewpoint to the concept of a complex tumor ecosystem, the involvement of TME in the tumor progression has received significant attention (71). As introduced earlier, components in TME are actively engaged in modulating tumor progression, and contribute to shaping of cancer metabolism landscape. In addition to intrinsic oncogenic factors, extrinsic regulation by TME factors is also important in reprogramming of lipid metabolism. Here, with a special focus on lipid metabolic pathways, we review the interaction of tumor cell and important factors of TME, including stress factors (hypoxia and acidosis), cellular (stromal cells) and molecular components (cytokines, metabolites) (**Table 1**).

**Table 1:** Non-exhausted list of microenvironmental factors regulating tumor lipid metabolism.

Factors	Cancer	Phenotype	Target	Reference
**Hypoxia**				
HIF-1	Ovarian cancer, hepatoma, glioblastoma, breast cancer	FA uptake ↑, LD accumulation ↑	FABP3/4/7 ↑	(72–74)
HIF-1	Breast cancer, hepatoma, cervix cancer	Endocytosis of lipoproteins ↑	LDLr ↑/VLDLr↑	(75)
HIF-1	Breast cancer	Reductive glutamine metabolism ↑→ lipogenesis↑	SNAT2 ↑, SIAH2 targeted ubiquitination and proteolysis of OGDH2↑	(76, 77)
HIF-1	Breast cancer	FAS ↑	AKT/mTOR/SREBP-1 ↑	(78)
HIF-1	Hepatoma	Cholesterol synthesis ↑	HMGCR ↑	(79)
HIF-1,2	Hepatoma, ccRCC	FAO ↓ Lipid accumulation ↑	β-oxidation enzyme: PGC-1α ↓ CPT1A ↓	(80, 81)
HIF-1	Clear cell renal cell carcinoma	FAO ↓	ACAD ↓	(82)
HIF-1α	Prostate cancer	Choline metabolism ↓	HIF-1-activated HRE7 within the promoter region of Chk→ Chk↓→ tCho ↓ PC ↓	(83)
HIF-2α	Clear cell renal cell carcinoma, hepatoma	FA desaturation ↑	SCD1 ↑	(84, 85)
HIF-1	Hepatoma, cervix cancer, renal carcinoma	LD formation ↑	TG biosynthesis enzyme ↑ AGPAT2 ↑ PLIN2 ↑ HIG2 ↑	(86–88)
HIF-1	Hepatoma, cervix cancer	Intracellular lipolysis ↓	HIG2 ↑ATGL ↓	(89)
Hypoxia	Breast cancer, prostate cancer	Acetate metabolism ↑	ACSS2 ↑	(90)
**Acidosis**
	Cervix cancer	FAO ↑ FAS ↑	Mitochondrial complex I activity ↓ histone deacetylation of ACC2 Histone deacetylation-mediated ACC2 repression	(53)
	Pancreatic cancer, cervix cancer, colon cancer	Acetate metabolism ↑ Cholesterol biosynthesis ↑	SREBP-2 ↑→ ACSS2 ↑ cholesterol biosynthesis enzyme ↑	(91)
	Cervix cancer, colon cancer	LD formation ↑	TGF-β2 signaling ↑→ CD36 ↑, DGAT1 ↑	(92)
	Glioblastoma	LD formation ↑	HSPG→ MAPK pathway→ lipoprotein uptake↑	(93)
	Prostate cancer	LD trafficking ↑	V-ATPase, PEDF	(94)
	Melanoma	Membrane remodeling	Unknown	(95)
	Mouse mammary carcinoma	Choline metabolism remodeling	Putative: glycerophosphocholine-diesterase enzyme ↓ phosphatidylcholine catabolism ↑→glycerophosphocholine ↑ PC ↓	(96)
**Nutrient deprivation**				
	Glioblastoma multiforme	Lipogenesis ↑	Lipid depletion → SREBP ↑→ maintain the expression of lipid biosynthesis genes → lipid biosynthesis → survival	(97)
	Breast cancer, prostate cancer	FA desaturation ↑	SCD1 ↑	(98)
	Breast cancer, prostate cancer,	Acetate metabolism ↑ lipogenesis ↑	Hypoxia and lipid deletion → ACSS2 ↑→ acetate uptake ↑ acetate contribution to lipogenesis ↑	(90)
	Leukemia, colon cancer, lung cancer	Profound alterations in classes of TG and CE	unknown	(99)
Autophagy	Solid tumors	Lipolysis and lipophagy of LD↑	mTORC1	(100, 101)
	Cervix cancer, hepatoma, osteosarcoma, colon cancer	LD formation↑→ protective lipid buffering system	DGAT1 ↑→ selectively channel autophagy-liberated FAs into new LDs, HIG2 ↑→ TG lipolysis ↓	(102, 103)
	LKB1-deficient KRAS-driven lung cancer	Maintain FAs level and prevent excessive FAO to survive energy crisis	Recycle intermediates to compensate for LKB1 loss	(104)
**Stromal cells**				
CAA	Ovarian cancer, breast cancer	lipid-dependent energy generation	Lipids derived from lipolysis in CAA, PPARγ→ CD36 ↑, FABP4 ↑ (OV)/FABP5 ↑ (BC)	(19, 105, 106)
	Breast cancer	Lipolysis ↑	ATGL ↑	(4)
Leptin	Breast cancer, colon cancer	FAO ↑	AMPK/PPARα pathway ↑ c-Myc/PGC-1 pathway ↑ JAK/STAT3 pathway ↑→ CPT1 ↑ c-Myc/PGC-1 pathway ↑ JAK/STAT3 pathway ↑ → CPT1B↑	(107–109)
	Breast cancer	Concomitant increase in FAO and FAS	Autophagy→ provide FAs for FAO, autophagy→ AKT signaling → SREBP-1/FASN ↑→ FAS ↑	(110)
	Breast cancer	Invasion ↑	PI3K/Akt/mTOR/SREBP2 ↑→ ACAT2 ↑	(111)
Adiponectin	Breast cancer	Lipid uptake ↓	CD36 ↓ LDLr ↓	(112)
Visfatin/ Resistin	Hepatoma	Lipogenesis ↑	FASN ↑	(113)
CAF	Pancreatic cancer, colon cancer, breast cancer	Lipogenesis ↑	Lipids/Alanine/LPC derived from CAF CD36 ↑ FATP1 ↑	(114–116)
CDE	Prostate cancer	Lipogenesis ↑	Glycolysis- and glutamine-dependent reductive carboxylation ↑	(117)
CDE	Gastric cancer	Lipid peroxidation ↓	miR-522→ arachidonate lipoxygenase 15 ↓→ lipid peroxidation ↓→ ferroptosis ↓	(118)
CD8^+^ T cell	Ovarian cancer treated with PD-L1 blockade	Lipid peroxidation ↑	Activated CD8^+^ T cell→ IFNγ→ SLC3A2 and SLC7A11 ↓→ glutathione peroxidase 4 ↓→lipid peroxidation ↑→ ferroptosis ↑	(119)
TAM	Ovarian cancer	Cholesterol uptake ↑	Membrane-cholesterol efflux ↑	(120)
EC	Colon cancer, Ovarian cancer	Cell membrane remodel	Glycerophospholipid ↑ PUFA ↑	(121)
**Cytokines**				
TNF-α	Hepatoma	Lipid accumulation ↑	AMPK pathway ↓ mTOR/SREBP-1 ↑, PPAR/ABCA1 ↑	(122)
	Cancer cachexia	Lipogenesis ↓	PPARγ pathway ↓	(123)
IL-17A	Ovarian cancer	FA uptake ↑	STAT3 signaling → FABP4 ↑	(124)
IL-15	Prostate cancer	FA uptake ↑	FABP1/4 ↑	(125)
IL-6	Colon adenocarcinoma	Fat loss in cancer cachexia	WAT lipolysis and browning ↑	(126)
TGF-β	Breast cancer	EMT→ lipogenesis ↓ FAO↑	SREBPs/ChREBP/FASN/ACC↓, AMPK ↑	(127, 128)
	Acidosis-adapted cancer cells	LD formation↑	Unknown	(92)
**Metabolites**				
Acetate	Breast cancer, Prostate cancer	Lipogenesis ↑	ACSS2 ↑→ acetyl-CoA pool, Epigenetic regulation of lipogenic genes (FASN)	(90, 129–131)
Butyrate	Colon cancer	Aerobic glycolysis ↓ glutamine metabolism ↑ lipogenesis ↑	AKT signaling→ GLUT 1 ↓ G6PD ↓, PKM2 ↑, PDK ↑ → glycolytic intermediates into TCA↓ glutamine metabolism ↑→ lipogenesis	(132–134)
LPA	Ovarian cancer	Lipogenesis ↑	HIF-1α ↑ AMPK ↓ SREBP ↑	(135, 136)
	Hepatoma	Lipid accumulation ↑	SCD ↑ TG synthesis and accumulation ↑	(137)
BCAA	Pancreatic cancer	Lipogenesis ↑	BCAA uptake ↑ → mTOR signaling↑→ SREBP↑, BCAT ↑ → mitochondrial biogenesis→ carbon source	(138–140)

### 3.1 Hypoxia

**Figure 2:**
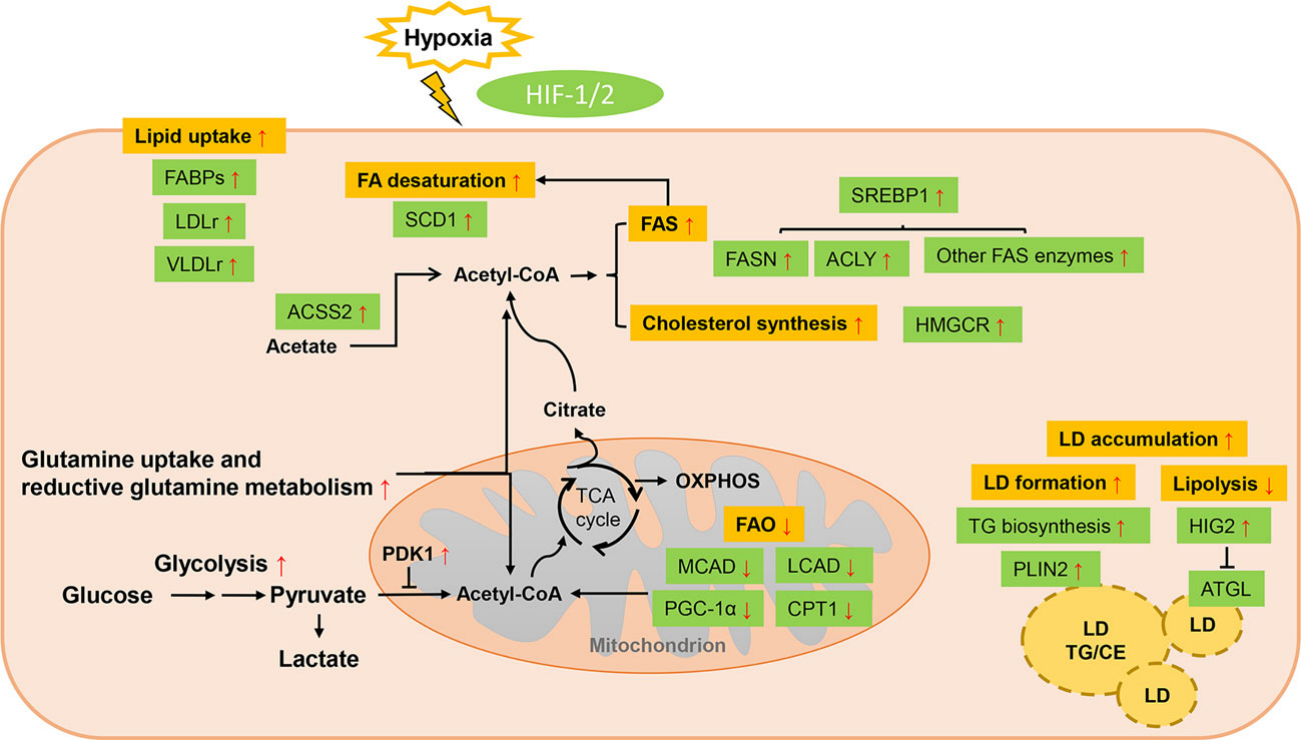
Lipid metabolism reprogramming of cancer cells under hypoxia. Response to hypoxia comprises a series of adaptive changes that are mainly regulated at the transcriptional level by the family of hypoxia-inducible factors (HIFs). HIF can induce a lipogenic cancer cell phenotype *via* enhancing lipid uptake, synthesis and storage while cutting down utilization. Uptake of exogenous lipids are promoted by enhanced expression of FABPs and LDLr/VLDLr. The conversion of glycolysis-derived pyruvate to acetyl-CoA is impaired due to the overexpressed PDK1 while ACSS2-mediated acetate metabolism and HIF-dependent stimulation of reductive glutamine metabolism function as important source of lipid precursors. *De novo* FA synthesis is favored by HIF-1 through upregulated SREBP-1, which enhance the expression of lipogenic enzymes. Cholesterol cynthesis is also enhanced with increased acitivity of HMGCR. Though the activity of SCD1 is restrained under hypoxia, HIF can elevate its expression to counteract this effect. Increased LDs accumulation and restricted lipolysis are other strategies employed by hypoxic cancers to avoid lipotoxicity. However, FA oxidation is inhibited by HIF through downregulating related enzymes.

The imbalance between the inadequate oxygen supply caused by abnormal vascularization and necessary oxygen consumption of tumor cells gives birth to the formation of a hypoxic environment, which is a common feature of solid tumors (141). Responses to hypoxia comprise a series of adaptive changes that are mainly regulated at the transcriptional level by the family of hypoxia-inducible factors (HIFs), which activate target genes involved in angiogenesis, metabolic reprogramming and other biological mechanisms (142). Despite the HIF-dependent regulation of carbohydrate metabolism is well established (143), the impacts of HIF on lipid metabolism have only recently been revealed: HIF can induce a lipogenic cancer cell phenotype *via* enhancing lipid uptake, synthesis and storage while cutting down utilization (144) (**Figure 2**).

FABPs (FABP3, FABP4 and FABP7) are upregulated in a HIF-1-dependent way to promote extracellular FA uptake and LD accumulation in various cancers (72, 73). Increased endocytosis of lipoproteins through upregulating the expression of LDLr and very low-density lipoprotein receptor (VLDLr) is another mechanism mediated by HIF-1 to promote lipid uptake in hepatoma, breast and cervix cancer cells (75).

Under hypoxic stress, HIF-1α-induced pyruvate dehydrogenase kinase isozyme 1 (PDK1) expression blocks the conversion of glucose-derived pyruvate to acetyl-CoA (145). To compensate, cancer cells adopt different metabolic mechanisms, such as HIF-dependent stimulation of reductive glutamine metabolism, or acyl-CoA synthetase short-chain family member 2 (ACSS2)-mediated acetate metabolism for alternative sources of FA precursors (76). In breast cancer, activation of HIFs upregulates the expression of glutamine transporters to enhance the uptake of glutamine and induces the gene that encoding the E3 ubiquitin-protein ligase SIAH2 to promote reductive carboxylation of glutamine-derived α-ketoglutarate to citrate for lipid synthesis (76, 77). Enhanced glutamine metabolism by HIF was also found in PDAC, RCC and lung cancer (146–148). Fatty acid biosynthesis is also favored by HIF-1 through modulating AKT/mTOR/SREBP-1 signaling in breast cancer, which upregulate the expression of lipogenic enzymes such as FASN (78). In HepG2 cells, HIF-1 was also found to increase the level and activity of HMG-CoA reductase (HMGCR) in the cholesterol synthesis pathway as well while the specific mechanism needs more elucidation (79). On the contrary, FAO is impaired due to HIF-1- and HIF-2-dependent downregulation of β-oxidation enzyme proliferator-activated receptor-γ coactivator-1α (PGC-1α) and CPT1A in hepatoma and ccRCC cells (80, 81). It is reported that in hepatoma cells HIF-1α could suppress FAO by repressing the expression of acyl-CoA dehydrogenases (ACAD) (82).

Hypoxia has also been shown to regulate the expression of Chk and the levels of total choline-containing compound (tCho) and phosphocholine (PC), which is attributed to the transcriptional control by HIF-1α through the binding of HIF-1α to hypoxia response element (HRE) sites in the regulatory promotor region of its target gene (83). In PC-3 prostate cancer cells, choline phosphorylation and the activity as well as expression level of Chk are decreased by hypoxia. This phenomenon was confirmed to be caused by activation of HRE7 within the Chkα promotor region by HIF-1α. HRE7 mutation could eradicate this effect (83). Yet another study in a human prostate cancer xenograft model reported contradictory results, represented by increased Chkα expression and elevated PC and tCho concentrations within hypoxic regions mediated by HIF-1α (149). Later it was shown that this Chk promotor-mediated upregulation takes place only when a highly repressive region, which contains the HRE7 site, is removed from the promotor (83).

The activity of SCD is constrained under hypoxic conditions since it is an oxygen-consuming enzyme and build-up of saturated FAs from enhanced lipogenesis could be toxic (150). However, HIF-2α can elevate the expression of SCD1 and induce the uptake of MUFAs to counteract this negative effect (84, 85). Increased LDs accumulation and restricted lipolysis are other strategies employed by hypoxic cancers to avoid lipotoxicity (47). HIF-1 directly upregulates the expression of enzymes involved in TG biosynthesis pathway and LD coat protein perilipin 2 (PLIN2) to favor LDs formation in cancers upon oxygen deprivation (86, 87). As both a LD protein and a direct target of HIF-1, hypoxia-inducible protein 2 (HIG2, also known as hypoxia inducible lipid droplet associated, HILPDA) functions in formation of LDs (88). Moreover, lipid accumulation is further supported by repressing intracellular lipolysis, which is mediated through HIF-1-mediated inhibition of adipose triglyceride lipase (ATGL) *via* HIG2 (89).

Many studies have shown that genetically or pharmalogically inhibiting HIF can revert the effects of lipid accumulation in various mouse models (151). HIF inhibitors are currently being tested, along with conventional therapies, for the treatment of different types of cancer (152). However, as the repertoire of direct HIF targets regulating of lipid metabolism involves complicated cascades, further studies are needed to unravel the exact steps controlled by HIFs to develop more precise anticancer treatments.

### 3.2 Acidosis

**Figure 3:**
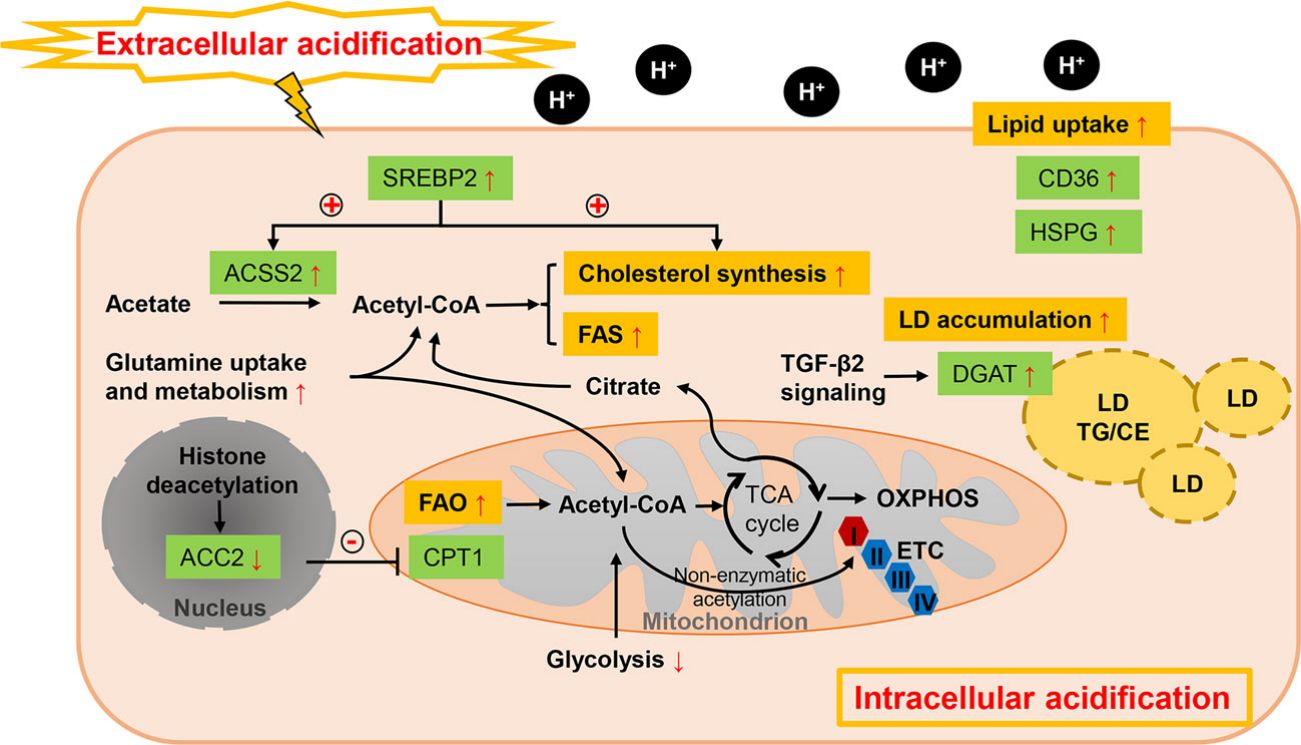
Lipid metabolism reprogramming of cancer cells under acidosis. While glucose-derived acetyl-CoA is dramatically reduced, FA metabolism is profoundly altered in response to ambient acidosis with a shift toward FAO as a main source of acetyl-CoA to support TCA cycle and downstream lipid synthesis pathways and exogenous uptake of lipids is also enhanced. Different with the hypoxia condition, FAO and FAS could co-exist in acidosis-adapted cancer cells, which is orchestrated by restraining the ETC complex I activity and repressing histone deacetylation-mediated ACC2. Cholesterol biosynthesis is also enhanced by pH-induced SREBP2 to maintain cellular cholesterol level under low pH. Meanwhile, acetate and glutamine act as critical substrates in acidic milieu. LD formation is favored in acidic pH *via* TGF-β2 mediated overexpression of DGAT1. “+” in circle means positive effects and “-” in circle stands for inhibition.

Acidosis, another hallmark of the TME, is the consequence of exacerbated glycolytic metabolism and CO_2_ hydration with a reduced removal of acidic waste products like lactate (153). It in turn impacts (independently from hypoxia) the metabolic preferences of cancer cells and contributes to the increased cancer aggressiveness (153). Evidence has shown that acidosis could induce metabolic rewiring far from restricted to inhibitory effect on glycolysis (154) (**Figure 3**).

While glucose-derived acetyl-CoA is reduced, FA metabolism is profoundly altered in response to ambient acidosis with a common shift toward FAO as a main source of acetyl-CoA to support TCA cycle and downstream lipid synthesis pathways (53). Increased bioactive acetyl-CoA from the stimulated oxidative flux has been suggested to restrain the electron transport chain complex I activity by non-enzymatic acetylation, preventing the overproduction of reactive oxygen species. Acidosis can also stimulate sirtuin-mediated nuclear histone deacetylation, thus repressing the ACC2 in the FAS pathway, whose product inhibits mitochondrial FAO (53). In parallel, FAS is supported by the reductive metabolism of glutamine under acidic pH (53, 155). Hence, FAO and FAS could paradoxically co-exist in acidosis-adapted cancer cells. Distinctive transcriptional regulation is also revealed in acidic extracellular pH (pHe) treated cancer cells from those of hypoxia and nutrient deprivation. SREBP2 is activated in various cancer cells lines by acidic pHe through enhancing its translocation and promotor binding to its targets, together with intracellular acidification (91). Cholesterol biosynthesis is therefore enhanced by pH-induced SREBP2 and cellular cholesterol level is sustained under low pH, while ACSS2-mediated acetate metabolism is also enhanced to provide growth advantage (91). It’s also interesting to note that cancer cells tend to exhibit a lipid-storing phenotype upon microenvironmental acidosis stimulation. Acidic pH promotes autocrine transforming growth factor-β2 (TGF-β2) signaling, which in turn favors the formation of LDs (92). Expressions of both CD36 and the final actor of FA accumulation as neutral lipids, diacylglycerol acyltransferase (DGAT1), respectively, are regulated by TGF-β2 produced by acidosis-adapted cancer cells (92). This LD-rich phenotype was also found in glioblastoma under hypoxic and acidic stress, where heparan sulfate proteoglycans (HSPG) could trigger the ERK/MAPK pathway and play an important role in hypoxia and acidosis-induced internalization of lipoproteins (93). LD trafficking in cancer cells can be also modulated by acid pHe. Upon lowering pH, maximum LD velocity and LD peripheral clustering was promoted in a V-ATPase- and lipolysis regulator pigment epithelium-derived factor (PEDF)-dependent manner (94). Except the influences on lipid amount, microenvironmental acidosis also changes the lipid composition such as structural changes of membrane phospholipids (95). Significantly increased glycerophosphocholine correlated with decreased PC was found in perfused mammalian tumor cells, which might attribute to the inhibition effects of acidic pH on glycerophosphocholine-diesterase enzyme, together with concomitantly activation of phosphatidylcholine catabolism (96).

Approaches for targeting acidosis have been raised such as neutralization buffers, acid-activatable agents, proton pump inhibitors and acidogenic metabolism inhibitors (156). Preclinical and some clinical studies have shown that targeting tumor acidity can improve anti-cancer therapy responses (156). Given the adaptions in lipid metabolism made by cancer cells under acidic environment, interfering with these metabolic vulnerabilities simultaneously with acidic pHe offers new perspectives for lipid-targeting therapy. For instance, to develop genetically or pharmacologically inhibit histone deacetylation of ACC2 to suppress FAO in acidosis-adapted cancer cells, or to identify enzymes in TGF-β2-induced LD formation whose activity is crucial at acidic pH.

### 3.3 Nutrient Deprivation

**Figure 4:**
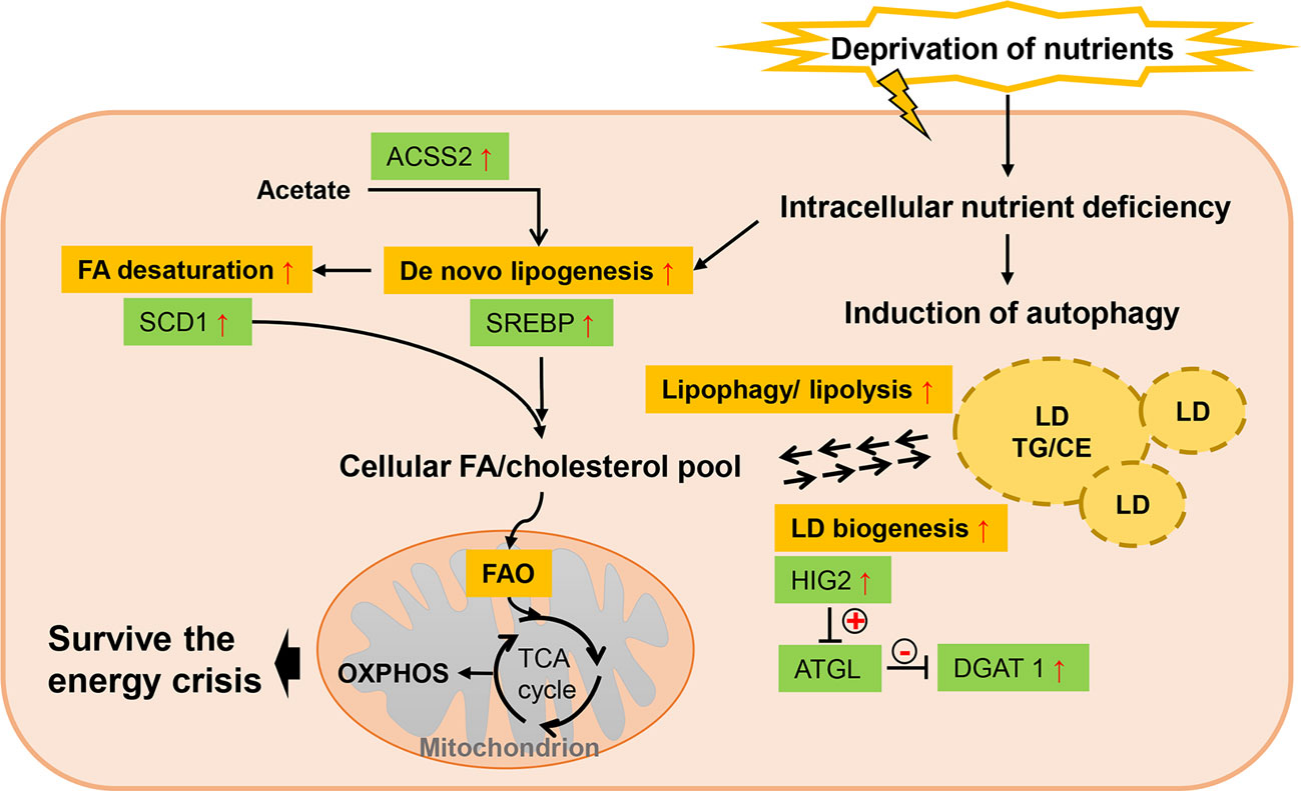
Lipid metabolism reprogramming of cancer cells under nutrient deprivation. Low serum condition allows cancer cells to be more reliant on *de novo* lipogenesis for FA acquisition rather than exogenous uptake, which is mainly favored by elevated SREBP. As exogenous MUFAs supply is restricted, SCD1 is upregulated to maintain FA desaturation. Nutrient deprivation can induce autophagy in cancer cells and LDs are turned over to generate FAs through cytosolic lipolysis and autophagy-mediated lipophagy. But LD biogenesis is also induced in response to high autophagic flux, providing a lipid buffering system to alleviate lipotoxic cellular damage in autophagy. “+” in circle means positive effects and “-” in circle stands for inhibition.

The poor blood perfusion also leads to limited availability of nutrients in TME, which allows alternative metabolic pathways other than glucose and glutamine metabolism to be mobilized. Under low serum condition, tumor cells are more reliant on *de novo* lipogenesis than the exogenous uptake for FA acquisition (90) (**Figure 4**). Transcriptional activity of SREBP is found to be induced by serum lipid depletion regardless of the oxygen status in glioblastoma multiforme, thus supporting cell survival (97). ACCSS2-mediated acetate metabolism, regulated by SREBP2, also contributes to cancer cell growth when exposed to oxygen and lipid withdrawal (90). Endogenous FA desaturation is increased in breast and prostate cancers by elevated expression level of SCD1 in low-serum conditions, where MUFAs supply is restricted (98). Moreover, nutrient deprivation can induce autophagy in cells, which is a catabolic process acutely sensitive to nutrient availability and recycles cellular constituents to generate primary substrates for energy production and survival (104). Though autophagy is important for most normal tissues, tumor cells exhibit particularly dependence on it for survival under stress, especially in RAS-driven tumors (100). Study revealed that autophagy compensated for tumor suppressor liver kinase B1 loss in KRAS-driven lung tumor cells to maintain levels of free fatty acids, while autophagy deficiency led to excessive FAO and energy crisis (104). Also, LDs are turned over to generate FAs through cytosolic lipolysis and autophagy-mediated lipophagy for FAO in response to nutrient starvation (100, 101). Intriguingly, LD biogenesis is also induced as response to mTORC1-regulated high autophagic flux during starvation (157). This seemingly counterintuitive process, *via* gathering autophagic-released FAs into new LDs, provides a lipid buffering system to alleviate lipotoxic cellular damage in autophagy (102, 157). And finally, these starvation-induced LDs are also lipolytically degraded (157). DGAT1 is required in this protective LD formation in tumors to selectively channel FAs into new LDs, which can be blocked by ATGL (157). Suppressing the ATGL activity, HIG2 is confirmed to be essential in LD biogenesis independently of HIF-1 transactivation (103). In addition to these effects on modulating cellular lipid amount, serum-deprivation significantly influences lipidomic profiles of tumor cells while no robust changes are observed under hypoxic condition (99). In this lipidomic study, cancer cells displayed profound alterations in TG compositions as well as decrease in cellular levels of all CE subspecies.

The essential role of starvation-induced autophagy in providing metabolic substrates to prevent energy crisis suggest promising application of autophagy inhibitors. But as basal autophagy is important for maintaining cellular homeostasis, the mechanisms for each tumor type and their dependence on autophagy need to be fully elucidated. It is required for utility of autophagy inhibition in combination with nutrient deprivation and to reduce side effects in normal tissues.

### 3.4 Stromal Cells

During formation of malignancies, tumor cells recruit stromal cells efficiently and educate them to form a suitable TME for progression as well as metastasis (158). These surrounding cells includes cancer-associated adipocytes (CAAs), cancer-associated fibroblasts (CAFs), immune cells and endothelial cells (ECs). The complex interplay between cancer cells and stromal cells leads to a reciprocal metabolic reprogramming of both cell types. Mounting evidences have illuminated that stromal cells, particularly CAAs and CAFs, critically modulate cellular lipid metabolism in solid tumors exploiting mechanisms like paracrine transfer of metabolites or non-cell-autonomous regulation of metabolic signaling pathways.

#### 3.4.1 Cancer-Associated Adipocyte and Adipokines

CAAs represent a vital source of lipids and adipocytokines, and their essential roles in driving metabolic reprogramming and facilitating tumor progression and drug resistance are increasingly appreciated, particularly in obesity-related cancers such as prostate, breast, ovarian and colon cancers (4, 5, 159, 160). These types of cancers are prone to growth and metastasis in an adipocyte-rich microenvironment. Thus, defining the dynamic crosstalk between CAA and tumor help to address the clinical challenges confronted by obesity-related malignancies. Secreted signaling molecules from cancer cells can trigger lipolysis in CAAs and degraded lipids are smuggled to the cancer cells in a way mediated by lipoproteins, exosomes and serum albumin (105). These lipids can either be stored in LDs or fueled by β-oxidation, indicating adipocytes as an energy provider in the TME. Apart from this, CAAs can induce metabolic switch of cancer cell from glycolysis to lipid-dependent energy generation. This was demonstrated in a number of co-culture studies that the uptake and mitochondrial utilization of fatty acids in cancer were significantly promoted by CAAs (19, 160). Co-culture of ovarian cancer cells with omental adipocytes upregulated CD36 expression and accelerated FFA uptake in cancer cells (19). Elevated expression level of FABP4 is identified to be a key event in regulating adipocyte-induced lipid responses of co-cultured ovarian cancer cells, including β-oxidation, lipid peroxidation and reactive oxygen species (ROS) generation (5, 106). Both CD36 and FABP4 are regulated by PPARγ signaling and CD36 is suggested as an upstream regulator of FABP4 (106). However in breast cancers, it is the elevated expression of FABP5 rather than CD36 or FABP4 that play critical roles in acceleration of FA trafficking and nuclear activation of PPARγ, along with induction of lipolysis and ATGL-mediated utilization of accumulated lipids (4).

Though the exact mechanisms underlying CAA-cancer cell crosstalk are unclear, it is acknowledged that secretion of adipocytokines and metabolic substrates contribute to the CAA-induced modulation. Leptin, a hormone mainly derived from adipocytes, shows pleiotropic effects on regulating energy balance and cancer progression (161, 162). Leptin has been known to boost FAO and OXPHOS for energy generation (107), whereas inhibit glycolysis and shift glucose catabolism towards biosynthetic pathways to provide intermediate precursors in breast and colon cancers (107, 108). It was reported that overexpression of CPT1 and upstream activation of AMPK/PPARα signaling pathway and c-Myc/PGC-1 pathway are involved in leptin-stimulated FAO (108). Moreover, leptin-activated JAK/STAT3 pathway also takes part in activating FAO through upregulation of CPT1B in breast cancer stem cells, which is critical for self-renewal and chemoresistance (109). Under physiological conditions, enhanced FAO is usually accompanied with reduced FAS. Consistently, researcher previously proved this in HCT116 and MCF-7 cells treated with leptin for 48 h, as decreased FASN expression together with increased FAO indicated (107). However, these two pathways can occur simultaneously in cancer cells under certain conditions, such as in previously mentioned acidosis-adapted cancer cells and breast cancer cells exposed to oxidative stress (51). A recent study has suggested that leptin drives a concomitant increase in FAO and FAS in MCF-7 cells, which is ascribed to the activation of autophagy (110). Specifically, leptin-activated autophagy enhances lipolysis and the release of free FAs, which further facilitates leptin-induced FAO for energetic production. Meanwhile, the expression of SREBP-1 is upregulated by leptin *via* autophagy and PI3K/AKT signaling pathway, resulting in overexpressed FASN and enhanced *de novo* lipogenesis. Researchers showed that inhibiting or silencing SREBP-1 or FASN impeded cancer cell viability promoted by leptin. Yet, the contradictory results of FASN expression in breast cancer indicated in these two studies need further verification to see whether the effect of leptin varies in time-dependent or dose-dependent manner (107, 110). Another study also revealed that leptin elevated expression of SREBP-2 *via* PI3K/AKT signaling, thus upregulating acetyl-CoA acetyltransferase 2 (ACAT2) to favor the migration and invasion of breast cancer cells (111). Taken together, leptin reconstructs cancer cellular lipid metabolism by multifactorial modulating biosynthesis and utilization of lipids, which further facilitate tumor progression.

Adiponectin is another adipocyte-specific hormone, yet elicits molecular effects opposed to leptin, as it may restrict tumorigenic processes. While identification and better understanding in the effect of adiponectin is cancer-specific lipid reprogramming still need further investigation, adiponectin is putatively considered as a negative modulator of lipogenesis. Adiponectin decreased lipid uptake through suppressing CD36 and LDLr expression in cancer cells (112, 163). As both an activator of AMPK and inhibitor of PI3K/AKT pathway, adiponectin is presumed to promote FAO and restrain lipogenic biosynthesis in cancer. Yet, this hypothesis requires corresponding proofs in the context of complicated signaling network of cancer cells. In addition, adipokines like visfatin and resistin also showed the potential to stimulate lipogenesis in liver cancer cells with elevated FASN expression (113). Inflammatory cytokines released by adipocyte, such as TNF-α, IL-6, are also able to affect cancer lipid metabolism with paracrine and endocrine activity, which will be discussed later.

#### 3.4.2 Cancer Associated Fibroblast

The role of CAFs, a major cellular component within tumor stroma, in the progression and spread of various cancers has been well-established (164). Rather than just being educated by tumor cells, CAFs have been suggested to play an active role in shaping tumor metabolism, including the lipid metabolic pathways (165). Through self-metabolic reprogramming, CAFs serve as hubs of lipids to support breast cancer cells growth with over-expression of FASN (114). FATP1 and CD36 are found as putative targets to disturb the smuggling of lipids between CAFs and breast cancer cells (114, 166). In addition to FAs, other CAF-derived metabolites like alanine are also utilized in pancreatic cancer cells as carbon sources for biosynthetic processes, including lipid synthesis (115). A recent study in PDAC cells showed that lysophosphatidylcholines (LPC) from CAFs cells supported membrane synthesis in cancer cells as well as the production of LPA by autotaxin (ATX) that was further engaged in paracrine activation of PI3K/AKT pathways (167). These CAF-derived metabolites and bioactive molecules are transported to tumor cells, at least partly, by CAF-derived exosomes (CDEs). Containing proteins, miRNAs and intact metabolites, CDEs are initially identified as a key component to support tumor growth under nutrient-deprived stress (117). CDEs could switch mitochondrial OXPHOS towards a glycolysis- and glutamine-dependent reductive carboxylation phenotype in cancer cells, thus replenishing for *de novo* lipogenesis (117). Interestingly, these metabolic alterations elicited by CDE-metabolite were independent of oncogenic KRAS in pancreatic cancer, suggesting CAFs’ ability to reprogram and support cancer cell metabolism independent of oncogene activation (117). The researchers later identified the glutamine anabolic pathway in CAF as a possible target to cause systemic metabolic vulnerabilities in ovarian cancers (168). Besides, miR-522 in the CDEs was found to suppress the lipid-ROS production in gastric cancer, *via* inhibiting the main mediator arachidonate lipoxygenase 15 (118). Ferroptosis, a form of cell death due to dysregulated membrane lipid peroxidation arising from an iron-dependent ROS accumulation, is thereby inhibited in gastric cancer, contributing to the tumor growth and decreased chemo-sensitivity to cisplatin and paclitaxel (118).

#### 3.4.3 Immune Cell and Endothelial Cell

As a crucial determinant of the phenotype and function of immune cells, metabolic reprogramming in immune cells caused by TME has been fairly defined and applied in manipulation of immune responses for cancer. Lipid metabolism alterations in immune cells also play a role in coordinating immunosuppression and tumor immune escape (169). However, only few researches described the effect of immune cells and their released factors on lipid rewiring of their neighboring cancer cells.

CD8^+^ T cells take a leading position in anti-tumor response among the tumor-infiltrated immune cells and activated effector T cells in the TME mainly depend on glycolysis and FAS, while memory T cells and CD4^+^ regulatory T cells maintain their functions by enhancing FAO (169). Metabolic competition by tumor cells restricts T cells and dampen their interferon gamma (IFNγ) production and other functions, thus facilitating tumor progression. A study reported that during immunotherapy, activated CD8^+^ T cell would enhance lipid peroxidation in tumor cells and finally led to ferroptosis. Mechanistically, it was illuminated that the IFNγ released from CD8^+^ T cells restrained uptake of cystine by tumors through downregulating the expression of SLC3A2 and SLC7A11, two subunits of the glutamate-cystine antiporter system x. This further inhibits glutathione synthesis and induces a decrease in glutathione peroxidase 4 expression, which is essential in the antiperoxidant defence, consequently promoting cell lipid peroxidation. Targeting IFNγ or iron-dependent lipid peroxidation pathway in combination with checkpoint blockade therefore provides new insights for combinatorial cancer treatment (119).

Growing evidence has shown that metabolic restriction imposed by tumor cells would restrict immune cell function thus promote immunoescape and caner progression (158, 169). In gastric adenocarcinoma, FAO-dependent tissue-resident memory T cells was outcompeted by cancer cells for lipid uptake. Blockade of PD-L1 would promoting lipid uptake and resulting better survival of Trm cells, *in vitro* and *in vivo* (170). However, a recent work indicated that the suppression of immune cells is not caused by cell-extrinsic nutrient competition, evidenced by the fact that the uptake of glutamine and lipids was dominated by cancer cells instead of by immune cell in TME (171, 172). Therefore, more researches are required to clarify this issue.

In most tumors, tumor associated macrophages (TAMs) adopt an ‘alternative’ phenotype (M2-like) exerting anti-inflammatory and pro-tumoral effects. Studies have proved that enhanced FAO, elevated expression of CD36 and accumulated lipids are essential in activation and differentiation in TAMs (173). In ovarian cancers, it’s uncovered that tumor cells can scavenge cholesterol from TAMs by promoting membrane-cholesterol efflux (120). IL-4 mediated signaling such as inhibition of IFNγ-induced gene expression is promoted due to the increased cholesterol efflux in TAMs, which is associated with pro-tumoral functions (120).

The ECs within tumor also undergo phenotype alteration to provide a promoting niche for cancer cells. An *in vitro* experiment reported that ECs triggered coherent and non-cell line specific increase in expression of both glycerophospholipid and poly-unsaturated fatty acids (PUFA) in cancer cells, which could be associated with remodeling of cancer cellular membrane to improve cellular cross-talk or modulate signaling pathways (121). But the underlying manipulation mechanism needs further explanation.

#### 3.4.4 Therapeutic Strategies Targeting Stromal Cells

Above mechanisms can be exploited therapeutically at the level of the stromal cells to impede tumor progression. Inhibition of lipogenesis or lipolysis in stromal cells and lipids uptake by malignant cells would be the most attractive approach to cut off lipid supply to tumor cells. However, these specific targeting strategies have not been validated in well-designed clinical trials and accompanying effects on other cells is a big challenge. Moreover, targeting CAAs alone is less likely to be effective since they are unlikely to lead to a complete reversal and ablation of tumor growth. Small-molecule inhibitors designed to target CAFs have also been developed, either by blocking the activation and trans-differentiation of stromal cells into CAFs, or silencing signaling pathways activated in CAFs and their downstream effectors (174). Blocking secretion and uptake of stroma-derived exosomes also represents a tantalizing target for clinical implementation to disrupt the level of *de novo* lipogenesis and oxidation. When targeting ECs, some methods are designed to normalize tumor vasculature, thus mitigating hypoxia and acidosis in TME and improving the efficacy of therapies (175). Overall, an integrated view of these cell-cell interactions needs to be further established for improved therapeutics and manageable adverse effects when simultaneously targeting multiple components of the TME.

### 3.5 Cytokines

Cytokines are messenger molecules secreted by diverse cell types, which plays a pivotal role in the intensive cross cell dialog in TME. Cytokines, like growth factors and interleukins (ILs), can either stimulate tumor progression or suppress tumor growth, generally depending on the context (176). This sparks a heated search for potent mechanisms and combination therapies, and the immunomodulatory and inflammatory potential of cytokines have been extensively explored in tumors. Here we focus on its impacts on lipid metabolism.

Tumor necrosis factor α (TNF-α), a multifunctional cytokine and adipokine, is primarily produced by adipocytes, activated macrophages and monocytes in the TME and can be induced by LPA (177). It is linked to direct induction of lipid accumulation in HepG2 cells, at least partially *via* the inhibition of AMPK and downstream activation of mTOR/SREBP-1 pathway (122). Similarly, exacerbated cholesterol accumulation in HepG2 cells is also attributed to TNF-α that interrupts cholesterol efflux through PPAR-regulated ATP binding cassette transporter A1 (ABCA1) pathway (178). IL-17A, an important pro-inflammatory cytokine produced by T helper 17 cells, could directly increase FA uptake in ovarian cancer, perhaps also in adipocytes, through upregulation of FABP4 instead of CD36 (124). STAT3 signaling is proved to be activated in this regulation, but not exclusively. IL-15 is found to enhance the expression of FABP1 and FABP4 in a prostate cancer murine model *via* gene expression analysis, but the possible pathway involved needs further investigation (125). Study also reveals that IL-6 might induce fat loss in cancer cachexia by regulating white adipose tissue lipolysis and browning (126). TNF-α as well play a negative role in modulating lipid biosynthesis and storage in cancer cachexia *via* downregulation PPARγ pathway (123).

TGF-β is a multipotent growth factor that could be highly secreted by cancer cells and surrounding stromal cells. As previously described, TGF-β signaling promotes FA uptake and TG accumulation into LD formation in acidosis-adapted cancer cells, favoring epithelial-to-mesenchymal transition (EMT) (92). However, the opposite observation has also been depicted. Down-regulated of FASN and ACC as well as decreased levels of carbohydrate-responsive element-binding protein (ChREBP) and SREBPs in non-small cell lung cancer are reported upon TGF-β-induced EMT (127). Moreover, FAO and mitochondrial OXPHOS are elevated in TGF-β1-induced EMT in MCF–7 cells through the AMPK pathway (128). These contrasting findings imply that TGF-β might play multifaceted roles in modulating lipid metabolism of cancer EMT, which would be determined in a context-dependent manner.

While the active participation of cytokines in inflammatory and immunity modulation receives considerable attention, their underlying impact on tumor metabolism is nonnegligible and is a worthy problem to probe for therapeutic cytokine applications. It’s hoped that novel combination approaches neutralizing inflammation, lethal metabolic alterations and immuno-suppression can improve the outcome of cytokine-targeted anti-tumor therapy.

### 3.6 Metabolites

#### 3.6.1 Microbiota Metabolites: SCFAs

The gastrointestinal (gut) microbiome has emerged as a pivotal microenvironmental factor for certain cancers, such as gastric cancer, colorectal cancer and HCC (179–181). The dysbiosis of gut microbiota diversity and related alterations in the level of gut microbial metabolites rewire the metabolic milieu and consequently contribute to the cancer progression. Dietary fiber is fermented by gut bacteria into short-chain fatty acids (SCFAs), including acetate, butyrate and propionate in a 3:1:1 stoichiometry (182). SCFAs are considered important substrates for energy metabolism, with propionate serving as a substrate for gluconeogenesis while acetate and butyrate serving as substrates for *de novo* lipogenesis.

Though acetate can also be acquired from deacetylation processes and ethanol metabolism, dietary food saccharolytic fermentation by intestinal microbiota is thought to be the main source of exogenous acetate (182). Intriguingly, acetate is specially positioned at the intersection of metabolism and genetics for its roles as both a carbon source for lipid biomass and an epigenetic regulator of post translational protein modification (129). Cancer cells exhibited activated cytosolic ACSS2-mediated acetate metabolism, which promotes tumor growth by replenishing the acetyl-CoA pool under hypoxia, acidosis and other stressed conditions (90). Consistent with this, radiolabeled acetate taken up by cancer cells has been shown to be greatly elevated than normal cells and mainly donates to the carbons in FAs under hypoxia (130). Moreover, uptake of radiolabeled acetate can reflect FASN expression levels and further the sensitivity to FASN-targeted therapy in prostate cancer cell lines as it reduces significantly with the presence of FASN inhibitors (131, 183). Therefore, [1-11C]-acetate PET based on these mechanisms is a promising non-invasive tool to diagnose cancers and even predict FASN-targeted therapy outcome and recurrence in clinical applications (184, 185). Beyond an alternative source of acetyl-CoA production, acetate is also implicated in initiating epigenetic regulation to lipogenic genes of FASN, for cancer cell survival under hypoxic stress (129). These might also provide new insights into FASN-targeted therapy.

Similarly, butyrate is mitochondrially oxidized to acetyl-CoA for lipid synthesis and represents the major energy source for normal colonocytes while cancerous colonocytes primarily undergo aerobic glycolysis (186). However, it reduces cancer cell growth while improves differentiation and this differential growth impact of butyrate on normal and neoplastic colonic cells has been known as the “butyrate paradox”. Butyrate in the glycolytic cancer cells is inefficiently metabolized and its accumulating in nucleus exerts antineoplastic effects, which are mediated, at least partially by inhibiting histone deacetylase (HDAC) activity and epigenetically regulating downstream target genes (186). The function of butyrate (either being oxidized or being a HDAC inhibitor) is related to its concentration, since low dose (<0.5 mM) meets the energy needs while high concentration (0.5~5 mM) leads to inhibitory outcome in *in vitro* experiments (187). With regard to its effects on cancer metabolism, though initially highly glycolytic, colon cancer cells can switch to a butyrate/glutamine-utilizing phenotype induced by butyrate, which is characterized by a lower production of lactate and raised incorporation of carbons derived from glutamine into lipids (132). Mechanistically, the expression of membrane glucose transporter 1 and cytoplasmic glucose 6 phosphate dehydrogenase decreased under the regulation of AKT signaling, leading to inhibited glucose metabolism and nucleotide synthesis and causing proliferation arrest (188). Besides, butyrate positively regulates the expression of M2-pyruvate kinase (PKM2) and PDK in colon cancer cells, significantly suppressing upstream glycolytic intermediates being diverted into biosynthetic pathway (132, 133). Thereby, butyrate contributes to acetyl-CoA pool directly or indirectly through stimulating glutamine utilization and favors a lipogenesis phenotype, as LD accumulation is observed in butyrate-treated colonic cancer cells (132). When considering butyrate as an anticancer agent, whether and how these metabolic altering contributes to the proliferation inhibitory impact of butyrate still needs further exploration, and cancer types other than colorectal cancer should also be further studied.

Given the facts that the increased risk of certain gastroenteric tumors is linked to alterations of gut microbiota species and reduced production of SCFAs, efforts have been made to elucidate the relation between dietary pattern and gut microbiota. Dietary interventions like high-fiber diets and the supplementation with polyunsaturated fatty acids, polyphenols and probiotics, which are known to regulate gut microbiota and generate SCFAs, have emerged as potential therapeutical strategy to prevent or to be used as adjuvants to conventional therapy (189). Of note, there is not a simple linear relationship between gut SCFA levels and individual dietary components or bacterial strains. Algorithms to predict individual responses to dietary and pharmaceutical interventions based on microbial metabolites is essential for any nutrition-based approaches. Alternative strategies such as increasing gut microbial production of beneficial metabolites and specific inhibitors for microbial pathways that produce harmful metabolites also has great potential in the long run.

#### 3.6.2 Oncolipids: LPA

LPA is a byproduct of the lipid biosynthesis pathway that presents at high level in several cancer patients. It functions as a growth factor stimulating oncogenesis and invasiveness through activating G protein-coupled receptors (190). It is extracellularly converted from stroma-derived LPC by a secreted lysophospholipase D, ATX. LPA has been shown to induce a glycolytic shift in cancer cells, while studies also reveal that it elicits pro-lipogenic actions in ovarian cancer by transcriptionally up-regulating SREBP-FASN and dephosphorylation of AMPK-ACC pathways, which is mediated by an LPA receptor LPA_2_ (135, 136). Noteworthy, LPA was shown to activate HIF-1α *via* inducing a pseudohypoxic response, thus further modulating metabolism alterations in ovarian cancer mediated by HIF-1α (135). ATX/LPA axis was also found to be involved in lipid desaturation, *via* stimulating SCD expression, neutral lipid liver deposition and TG accumulation in HepG2 cells (137). Although it still remains to be confirmed whether ATX/LPA can have the same effect in other cancer types, the ATX/LPA axis, communicating with other lipid modified signaling, is engaged in lipid metabolism in malignant cells and is emerging as a novel cancer hallmark.

#### 3.6.3 BCAA

Branched chain amino acids (BCAAs; leucine, isoleucine and valine) are essential amino acids that are only available from dietary protein rather than from endogenous synthesis. The increase in plasma BCAAs has been associated with pancreatic cancer risk and cancer cells have exhibited increased BCAA uptake as well as over-expressed BCAA aminotransferase (BCAT), for proliferation (191, 192). BCAA metabolism in PDAC cells has been unveiled as critical carbon source for FA synthesis, as the knockdown of BCAT leads to dramatic reduction in levels of FAs and TGs, as well as the inhibition of tumor cell proliferation (138). Mechanistically, elevated BCAAs selectively activates mTOR signaling and triggers phosphorylation of downstream effectors, including SREBPs (139). BCAT also promotes mitochondrial biogenesis and function in a mTOR-dependent manner (140). Thence, disruption of the BCAA level or targeting pivotal enzymes indicates an exploitable therapeutic strategy for cancer therapy. However, as the most straightforward strategy, controlling dietary BCAA intake shows controversial effects in cancer therapy (193–197). An integral investigation is therefore required to find out whether this method has benefits for certain types of cancer.

## 4 Conclusions and Future Perspectives

The indisputable dependence on lipids for carcinogenesis and progression emphasizes a lipid metabolic plasticity of the malignant cells that may be employed at multiple molecular levels as therapeutic targets, especially in obesity-related cancers. Current preclinical and clinical studies have shown that a variety of compounds targeting enzymes and/or signaling involved in lipid metabolism show promising antineoplastic effects (198). However, side effects resulted from incomplete knowledge of complicated regulatory mechanisms of lipid metabolism are big challenges ahead. In addition to oncogenic events, TME is another critical player that has an emerging role in the shift of lipid metabolic pathways. Here we summarize some key factors in the TME, including stress factors (hypoxia, acidosis and nutrient deprivation), cellular factors (tumor associated stromal cells) and molecular factors (cytokines and metabolites) and their influence on lipid rewiring. Still, continued efforts are needed to determine all the underlying microenvironmental factors and their interactions. In this way, it will be possible to better correlate altered lipid profiles with changing microenvironment and to better explore the potential of lipid metabolism as an anticancer approach. Also, detailed mechanisms must be further completed to achieve the desired effect and avoid negative impacts in normal metabolic functions.

## Author Contributions

HW and LC conceived the manuscript. LC and MY wrote the manuscript. HW and MY provided comments. All authors contributed to the article and approved the submitted version.

## Funding

This work has been supported in part by Zhejiang Provincial Natural Science Foundation of China (LY17H160036), the Fundamental Research Funds for the Central Universities (2017FZA7010) and China Natural Sciences Foundation project (81301707), all to HW.

## Conflict of Interest

The authors declare that the research was conducted in the absence of any commercial or financial relationships that could be construed as a potential conflict of interest.

## Publisher’s Note

All claims expressed in this article are solely those of the authors and do not necessarily represent those of their affiliated organizations, or those of the publisher, the editors and the reviewers. Any product that may be evaluated in this article, or claim that may be made by its manufacturer, is not guaranteed or endorsed by the publisher.

## Glossary

**Table:**

α-KG	α-ketoglutarate
ABCA1	ATP-binding cassette transporter A1
ACAD	Acyl-CoA dehydrogenase
ACAT	Acetyl-CoA acetyltransferase
ACC	Acetyl CoA carboxylase
ACLY	ATP citrate lyase
ACSL	Acyl-CoA synthetase long chain
ACSS2	Acetyl CoA synthetase 2
AGPAT	Acylglycerophosphate acyltransferase
ACST	Alanine–serine–cysteine transporter
AMPK	AMP-activated protein kinase
ATGL	Adipose triglyceride lipase
ATX	Autotaxin
BCAA	Branched-chain amino acid
BCAT	BCAA aminotransferase
CAA	Cancer-associated adipocyte
ccRCC	Clear-cell renal cell carcinoma
CAF	Cancer-associated fibroblast
CE	Cholesterol ester
Chk	Choline kinase
CPT	Carnitine palmitoyltransferase
DGAT	Diacylglycerol acyltransferase
EC	Endothelial cell
ER	Endoplasmic reticulum
ETC	Electron transport chain
FA	Fatty acid
FABP	Fatty acids binding protein
FAO	Fatty acid oxidation
FAS	Fatty acid synthesis
FASN	Fatty acid synthase
FATP	Fatty acid transport protein
HCC	Hepatocellular carcinoma
HDAC	Histone deacetylase
HIF	Hypoxia-inducible factor
HIG2	Hypoxia-inducible protein 2
HMGCR	3-hydroxy-3-methylglutaryl-CoA reductase
HMGCS	3-hydroxy-3-methylglutaryl-CoA synthase
HSPG	Heparan sulfate proteoglycan
LCAD	Long-chain acyl-CoA dehydrogenase
LD	Lipid droplet
LDLr	Low-density lipoprotein receptor
LKB1	Liver kinase B1
LPA	Lysophosphatidic acid
LPC	Lysophosphatidylcholine
LXR	Liver X receptor
MAPK	Mitogen-activated protein kinase
mTOR	Mammalian target of rapamycin
MCAD	Medium-chain acyl-CoA dehydrogenase
MUFA	Monounsaturated fatty acid
OGDH2	2-oxoglutarate dehydrogenase
OXPHOS	Oxidative phosphorylation
PDAC	Pancreatic adenocarcinoma
PC	phosphocholine
PDC	Pyruvate dehydrogenase complex
PDK	Pyruvate dehydrogenase kinase
PEDF	Pigment epithelium-derived factor
PGC-1α	Peroxisome proliferator-activated receptor γ coactivator 1α
PKM2	Pyruvate kinase M2
PLIN2	Perilipin 2
PPAR	Peroxisome proliferator-activated receptor
PUFA	Polyunsaturated fatty acid
SCD	Stearoyl-CoA desaturase
SFA	Saturated fatty acid
SIAH2	Siah E3 ubiquitin protein ligase 2
SNAT2	Sodium-coupled neutral amino acid transporter
SREBP	Sterol regulatory element binding transcription factor
TAM	Tumor-associated macrophage
TCA	Tricarboxylic acid cycle
tCho	Total choline-containing compound
TG	Triacylglycerol
TGF-β2	Transforming growth factor-β2
TME	Tumor microenvironment
VLDLr	Very-low-density lipoprotein receptor
WAT	White adipose tissue
